# Outcomes of the Rope Skipping ‘STAR’ Programme for Schoolchildren

**DOI:** 10.1515/hukin-2015-0024

**Published:** 2015-04-07

**Authors:** Amy S. Ha, Angus Burnett, Raymond Sum, Nikola Medic, Johan Y. Y. Ng

**Affiliations:** 1 The Chinese University of Hong Kong, Hong Kong.; 2 Aspetar Orthopaedic and Sports Medicine Hospital, Qatar.; 3 Edith Cowan University, Australia.

**Keywords:** moderate-to-vigorous physical activity, school-based intervention, accelerometry, health-related quality of life, rope skipping, recess

## Abstract

Physical activity in children and adolescents is on a decline trend. To this end, we conducted a matched-pair randomized controlled trial to examine the effects of a 4-week STAR (School-based; Train-the-trainer; Accessibility of resources; Recreational) skipping programme. 1,386 schoolchildren from 20 primary and secondary schools were recruited. Schools were randomized into the experimental or wait-list control group. Participants self-reported their health-related quality of life using the KIDSCREEN-27. Accelerometers were used to measure the time a subgroup of participants (n = 480) spent in moderate-to-vigorous physical activity during school hours on five consecutive days. Measures were taken at pre- and post-test. At post-test, students in the experimental group, compared to those in the control group, engaged in less moderate-to-vigorous physical activity during school hours. Health-related quality of life from two groups of students was similar, but the experimental group reported higher levels of autonomy and parent relationships. Results suggested that although the intervention did not increase students’ physical activity levels, it slightly improved their health-related quality of life. Future studies should explore personal factors that might mediate the effect of the intervention.

## Introduction

Age-related decline in physical activity (PA) among children and adolescents has been reported (Bélanger et al., 2009). As with their Western counterparts, similar trends of declining PA levels across the lifespan have been reported amongst Asian youth (Ha et al., 2009). Low levels of PA may be the result of reductions in multiple domains, such as physical education, organized sports and travelling (van Sluijs et al., 2007). The amount of time spent on organized sports (Buliung et al., 2009) and free playtime (Sirard and Slater, 2008) has also decreased. Although much research has investigated the intensity of exercise and PA of children, researchers have advocated that health-related quality of life (HRQoL) should also be investigated in order to fully understand people’s physical, psychological and social aspects of well-being and function as perceived by themselves (Wallander et al., 2001).

To counteract low levels of PA, several strategies have been suggested. One approach that has shown some success consists of school-based interventions (Kriemler et al., 2011; Sallis et al., 2001; van Sluijs et al., 2007). This seems a logical setting to intervene as children and adolescents spend most of their day at school. Schools are also venues where students are constantly surrounded by other students, hence there is regular interaction with peers, providing social support to engage in PA. Researchers found that such interventions could lead to increases in school-based PA, as well as out-of-school PA (Kriemler et al., 2011). Opportunities to increase PA in school include time before school lessons as well as recess (Ridgers et al., 2006, 2011). Recess has been of particular interest as previous research has shown that children conduct around 50% of their school-based PA during this period (Escalante et al., 2014), and may gain between 5–40% of recommended daily PA during this time (Ridgers et al., 2006). Therefore, strategies targeted to increase students’ PA during recess are needed.

In the search for better methods to increase PA levels in schoolchildren, researchers have also turned their attention to school policy and the environment of the school playground (Bocarro et al., 2012). Strategies such as providing increased space in which to play, and using competent supervisors to promote PA have been suggested (Cardon et al., 2009). Moreover, strategies such as altering playground environments by providing markings on the ground and walls, or providing additional equipment have been investigated but have provided contrary results (Cardon et al., 2009; Haug et al., 2010; Verstraete et al., 2006). Evidence in the literature is also lacking in terms of the effectiveness of strategies needed to increase PA levels in schools with limited space and resources. This is of particular relevance to Asian countries characterized by a high population density outside of the school environment; furthermore, school playgrounds are also relatively small (Johns and Ha, 1999). Therefore, innovative strategies are required to address these limitations. Moreover, students from lower socioeconomic areas are at an increased risk of obesity (Stamatakis et al., 2009). Inexpensive solutions applicable for this population are hence required.

One solution that can potentially address the issue of physical space limits and factors related to low socioeconomic status is rope skipping. Rope skipping is a popular form of PA in some Asian countries and it has many potential advantages. Specifically, the related equipment is of low cost and requires little space. Moreover, rope skipping is a high intensity activity (Laurson et al., 2008; Town et al., 1980). Potentially, this means that students could spend more time in MVPA during recess (Chow et al., 2008). The present study was designed to test the hypothesis that schools with limited but adequate space, facilities, equipment, supervision and appropriate personnel (trained coaches and skipping ambassadors) could stimulate students to be physically active in school during recess. We also examined the extent to which the intervention affected HRQoL in schoolchildren. Based on the above, a randomized controlled trial was conducted. The aim of this study was to determine whether the intervention, delivered to students enrolled at primary and secondary schools and located in low-to-medium socioeconomic areas, was effective in increasing students’ PA levels during school time, and also improving their HRQoL. We hypothesized that students who received the intervention, compared to those who did not, would show higher levels of MVPA, measured using accelerometers, during five consecutive schooldays. We also hypothesized that students would report higher KIDSCREEN-27 scores (a measure of HRQoL) in schools receiving the intervention, compared to those from the control group.

## Material and Methods

### Procedures and Participants

Subjects in this study were a subsample of primary and secondary schoolchildren that participated in a larger study (*N* = 7,000 from 36 schools) called the Coca-Cola Rope Skipping STAR Programme. Identical measures for HRQoL and MVPA were taken at pre and post-test, which were eight weeks apart. A total of 20 schools (10 primary and 10 secondary) were included in the study. After pre-test measures were taken, participating schools were matched in ten pairs according to five matching variables, namely socioeconomic status, co-educational school setting, class size, student’s personal PA space during PE lessons and/or recess periods, and whether the school had an active recess policy. For each pair, the toss of a coin was used to randomize schools into either the experimental or a wait-list control group. Subjects’ parents/guardians provided written consent for students’ participation in the study. To ensure they were sufficiently physically healthy to engage in PA, all students were required to complete the Physical Activity Readiness Questionnaire (PAR-Q), also signed by the parent/guardian, before the start of the study. The protocol and all instruments used in the study were reviewed and approved by the Survey and Behavioural Research Ethics Committee at the Chinese University of Hong Kong.

A total of 1,592 students completed the KIDSCREEN-27 questionnaire at pre-test. Out of these participants, 1,386 (754 allocated to the experimental group; mean age = 12.03, *SD* = 1.84; 636 males, 662 females, 88 did not report) students also completed the post-test questionnaire eight weeks later. Only data from these participants were included in the analyses. The PA levels of a subsample of students (*n* = 495) were measured using accelerometers. In attempt to ensure this subsample was randomly chosen, the participants were selected using pre-assigned class numbers provided by the research team. Again, PA levels of 480 (237 in the experimental group; mean age = 12.66, *SD* = 1.90; 216 males, 238 females, 26 did not report) students were measured at pre and post-test.

### Intervention

The intervention provided in this study was called the STAR project – a School-based intervention; Train-the-trainers (PE teachers, student sports leaders, skipping coaches and ambassadors); Accessibility of resources (ropes and activity zones); and Recreational physical activity (recess and lunch periods). Based on the ecological model of health behaviour (Sallis et al., 2008), the proposed intervention was specifically designed to intervene at the social (i.e., introducing sports leaders and skipping ambassadors) and environmental (i.e., providing free ropes and starting a rope skipping corner) levels. Details of the STAR programme and the associated intervention are provided in Table 1. Essentially, the intervention involved a 12-hour skipping workshop spanning three days, which was held to support PE teachers and their student sports leaders to learn how to promote skipping in school settings. In addition, skipping ambassadors (professionally trained individuals) were also made available and helped participating schools conduct the relevant skipping activities. A 4-week school-based skipping programme was designed. Participants in the experimental group received a free package containing skipping materials, ropes, professional skipping training and ambassadors’ support during the research period whilst the control (delayed intervention) group only received skipping ropes. The research team also helped schools set up a rope skipping corner during recess and lunch periods, where skipping ropes and relevant materials were made available to all students.

### Measures

*Physical Activity.* ActiGraph GT3X+ accelerometers were worn by participants on five consecutive school days (Monday to Friday). Each morning children were given a numbered accelerometer to be worn by a teacher. Prior to the actual data collection, the teacher attended a familiarization session where they become acquainted with the monitors and how to wear them. The same teacher showed participants how to manage the accelerometers on the first day of pre-test. Participants were asked to wear the accelerometers during five schooldays and follow their normal daily school routine. They were asked to return the accelerometers to the teacher-in-charge for proper storage before the participants left their schools on each day. Participants wore the same device on all five days within each time point. Participants’ accelerometer data were considered valid only if they wore the accelerometers for two or more days. The cut-off values proposed by Evenson et al. (2006) were used as criteria for MVPA. Mean minutes spent in MVPA per day were used as the measure for PA. Previous studies have employed similar strategies to examine students’ PA during school hours (Nettlefold et al., 2011; Verstraete et al., 2006).

#### Health-related Quality of Life Questionnaire

The KIDSCREEN-27 (The KIDSCREEN Group Europe, 2006), a measure for five dimensions (i.e., Physical Well-Being, Psychological Well-Being, Autonomy & Parent Relation, Peers & Social Support, and School Environment) of generic HRQoL, was administered to participants. The KIDSCREEN items were responded using 5-point Likert scales (1 = *never* to 5 = *always*) of certain behaviours or the intensity of an attitude (1 = *not at all* to 5 = *extremely*). The recall period was one week. The original English KIDSCREEN-27 was translated into traditional Chinese (Cantonese) for the purpose of this study. Items were translated into Chinese using a back translation protocol (Van Widenfelt et al., 2005). Before administrating to participants in the current trial, the Chinese items were administered to a separate sample of 1,451 students in a pilot study. In this pilot study, subscale scores were found to be internally consistent (α = .81 to .87). Factorial validity of scale scores was also examined using confirmatory factor analyses, and the expected 5-factor structure was supported: χ^2^ = 1676.12, *p <* .01, CFI = .94, TLI = .93, RMSEA = .06).

### Statistical Analysis

Analysis of covariance (ANCOVA) was used to test our hypothesis regarding group (experimental versus control) differences in PA levels. Previous research has shown that there are sex and age differences in PA (Trost et al., 2002). Therefore, sex and age of participants was included as an independent variable and a covariate, respectively. To control differences in baseline, pre-test scores were included as covariates in the analyses. Similarly, we conducted five (one for each subscale) two-way (group x sex) ANCOVAs, with age and baseline scores as covariates, for KIDSCREEN-27 subscale scores at post-test.

## Results

### Preliminary Analyses

Descriptive statistics of measured variables at pre- and post-test are presented in Table 2. We first examined whether the intervention and control groups had similar levels of MVPA at pre-test. We found that students in the experimental group were more active than students in the control group, *F*(1,449) = 6.75, *p <* .01, partial η^2^ = .015. Consistent to findings with previous research (Trost et al., 2002), we also found that male students had higher levels of MVPA compared to females, *F*(1,449) = 49.68, *p <* .001, partial η^2^ = .100. However, contrary to previous findings (Trost et al., 2002), MVPA was positively associated with students’ age in our study, *r* = .16, *p <* .001. These variables were included as independent variables or covariates in our main analyses.

In terms of baseline scores of KIDSCREEN-27 subscales, we found sex differences in the Physical Well-Being (*F*[1,1295] = 21.46, *p <* .001, partial η^2^ = .016), and Autonomy & Parent Relation (*F*[1,1287] = 6.21, *p <* .05, partial η^2^ = .005) subscales. Specifically, females reported higher levels of physical well-being, while males had higher scores for perceived autonomy and parent relation. Age was also negatively associated with all KIDSCREEN-27 subscale scores (*r* = −.25 to −.07, *p <* .05), suggesting that older students reported lower HRQoL.

### Physical Activity

A two-way (group x sex) ANCOVA with age and baseline values as covariates was conducted. There was a significant main effect for group: *F*(1,448) = 15.30, *p* < .001, partial η^2^ = .033. Unexpectedly, the control group had higher levels of MVPA compared to the experimental group. The main effect for sex was not significant: *F*(1,448) = 0.93, *p* = .34, partial η^2^ = .002). The group x sex interaction effect was also not significant: *F*(1,448) = 0.67, *p* = .41, partial η^2^ = .002.

### Health-Related Quality of Life

For Physical Well-Being subscale scores, a main effect for sex was found, *F*(1,1288) = 6.14, *p* < .05, partial η^2^ = .005. Specifically, male students reported higher physical well-being than female students. However, the main effect for group was not significant: *F*(1,1288) = 0.34, *p* = .95, partial η^2^ = .001. In terms of the Autonomy & Parent Relation subscale, a main effect for group was found, *F*(1,1265) = 4.55, *p* < .05, partial η^2^ = .004, with students in the experimental group reporting higher scores compared to those in the control group. The main effect for group was not significant for the subscales of Psychological Well-Being (*F*[1,1290] = 1.27, *p* = .26, partial η^2^ = .001), Peers & Social Support (*F*[1,1287] = 0.31, *p* = .58, partial η^2^ = .000), and School Environment (*F*[1,1287] = 0.06, *p* = .81, partial η^2^ = .000). All other main and interaction effects examined were not significant.

## Discussion

In this study, we examined the effectiveness of the STAR intervention in terms of enhancing students’ MVPA and their HRQoL. The intervention was based on rope skipping, which is an activity of moderate-to-vigorous intensity (Laurson et al., 2008), but requires little space to participate in. These characteristics of the activity may be of relevance to the typical school environments in Asian countries. Using a matched-pair randomized controlled trial, the effectiveness of the intervention was examined. Contrary to our hypotheses, we found that students in the control (i.e., delayed intervention) group engaged in more MVPA compared to those in the experimental group at post-test, after controlling for pre-test scores. We did, however, find that students in the control group also had engaged in more MVPA at baseline. This suggested that perhaps students in the control group were more active in general, and therefore, they showed higher levels of MVPA, even after adjusting for baseline scores. Nevertheless, the effect size of this difference was very small. In terms of differences in HRQoL, the only group differences we found were related to students’ perception of their autonomy and relationship with parents. Again, the size of the effect was very small.

We examined how factors pertaining to interpersonal and environment levels within the ecological model of health behaviour (Sallis et al., 2008) might affect PA levels of students. Our results suggested that the intervention did not increase students’ PA levels. Although the intervention was designed based on these levels of factors, students’ perceptions of these factors were not measured. Therefore, the current investigation was unable to examine the mechanisms in which PA was supported (or not), and if any mediators existed. For example, future studies may measure participants’ perceptions towards social support from friends, sports leaders, ambassadors, or teachers in terms of their rope skipping participation. Measures for the perceived supportiveness of the school environment and/or the rope skipping corner should also be examined. Such measures will help researchers identify any underpinning mediators of the intervention. Apart from measurement issues, the intervention designed was aimed at increasing students’ PA through supporting the social and school environments. Factors at other levels within the ecological model (Sallis et al., 2008) could also be verified. For instance, personal level (e.g., self-efficacy or motivation towards PA) factors were not specifically enhanced or measured in the current study. Researchers have previously found that these factors predicted PA behaviours and also students’ quality of life (Standage et al., 2005; Trost et al., 1997). Under the framework, factors pertaining to school policies, such as whether PA was considered important by school principals and teachers, and the amount of resource schools put into supporting students’ PA should also be examined. Also, Zhang and Solmon (2013) proposed integrating an ecological approach with self-determination theory (Deci and Ryan, 2000), a theory of motivation, to simultaneously examine multiple levels of environmental, social and personal factors that may be related to students’ engagement in PA. This approach can be employed in future research.

Due to the limited space at homes and surrounding areas, schools in some Asian countries are important venues for students’ engagement in PA (Johns and Ha, 1999). In this study, we found that students averaged only 10 to 15 minutes of MVPA per day during school hours. That is, students only engaged in one sixth to a quarter of the daily MVPA recommended by the World Health Organization (2010) for children and adolescents (i.e., 60 minutes per day). This finding is of concern, as it suggests that most participants in the study are unable to reach the recommended guidelines for PA. Researchers should continue to devise interventions to address the problem of physical inactivity and sedentary behaviours in students attending schools of limited space. Although the STAR intervention we proposed was not effective in terms of promoting higher levels of PA in participants, rope skipping is still an activity that may be promoted given environment restrictions. Future interventions may aim to incorporate more diverse types of rope skipping activities in order to make the activity more interesting to students, and hence might facilitate their engagement in rope skipping, or more generally in PA.

Another limitation of the study is that MVPA was measured only during school hours, and it is unclear how much students engage in PA after they leave school premises. Previous research has shown that school-based interventions may also increase students’ PA levels out-of-school (Kriemler et al., 2011). As leisure-time PA also plays an important part in students’ total PA, for this reason future studies should also examine students’ PA levels after school and during weekends.

## Practical Implications

Researchers (Sallis et al., 2001, 2008) have proposed using school-based interventions to facilitate students participation in PA. Our proposed intervention, which mainly focused on creating an environment that facilitates PA, was not effective in promoting actual PA levels and HRQoL of students. The findings of the present study suggest that for a programme to be effective in promoting school health, it may also need to intervene at other levels within the ecological model.

## Figures and Tables

**Table 1:** Details of the STAR Intervention

Component	Participants	Duration	Details
1. Rope skipping workshops and day camp, led by professional coaches	33 PE teachers and 89 student leaders	12 hours	Training on how to promote rope skipping at school was provided
2. Rope skipping programme (intervention), by PE teachers, student leaders, ambassadors, and coaches	500 children aged 9 to 16 years, drawn from 10 matched pairs based on school variables	4 weeks	Experimental group: A rope skipping programme embedded with school PE curriculum and during recess periods was provided. Participants received a free package containing rope skipping teaching materials, ropes, professional coaching and ambassadors’ support. Wait list control group: Schools implemented their PE classes as planned but provided with free ropes only during school recess periods in order to increase students’ physical activity levels; no professional training and other relevant supports on skipping teaching. A delayed intervention was provided
3. School active recess	PE teachers and students from schools in the experimental group	10–15 minutes during school recess periods, over 3 months	Schools received a full set of materials including teaching manuals with DVD and students handbooks on rope skipping and nutrition to promote a healthy lifestyle to students and peers. Rope skipping corner were set up by schools providing space and ropes for students to skip during recess, lunchtime and after school

**Table 2:** Descriptive Statistics of Participants in the Intervention and Control Groups

Variable	Baseline			Follow-up		
	Experimental	Control	α	Experimental	Control	α
*Accelerometry statistics* (*n =* 480)						
Moderate-to-vigorous physical activity (min per day)	13.12 ± 8.31	15.87 ± 7.38	-	10.96 ± 8.34	14.93 ± 7.54	-
*KIDSCREEN* (*n* = 1,273)						
Physical Well-Being	3.62 ± 0.72	3.59 ± 0.76	.81	3.62 ± 0.76	3.65 ± 0.76	.83
Psychological Well-Being	3.60 ± 0.73	3.65 ± 0.78	.87	3.63 ± 0.72	3.70 ± 0.76	.88
Autonomy & Parent Relation	3.80 ± 0.81	3.86 ± 0.84	.83	3.78 ± 0.81	3.73 ± 0.86	.85
Peers & Social Support	3.34 ± 0.77	3.38 ± 0.79	.86	3.40 ± 0.77	3.45 ± 0.83	.87
School Environment	3.58 ± 0.80	3.63 ± 0.83	.87	3.60 ± 0.78	3.64 ± 0.85	.88
